# Pneumatic actuator and flexible piezoelectric sensor for soft virtual reality glove system

**DOI:** 10.1038/s41598-019-45422-6

**Published:** 2019-07-18

**Authors:** Kahye Song, Sung Hee Kim, Sungho Jin, Sohyun Kim, Sunho Lee, Jun-Sik Kim, Jung-Min Park, Youngsu Cha

**Affiliations:** 1 Center for Intelligent & Interactive Robotics, Korea Institute of Science and Technology, Seoul, Republic of Korea; 2 Department of Biomedical Engineering at Korea University, Seoul, Republic of Korea; 3 Department of Mechanical System and Design Engineering at Seoul National University of Science and Technology, Seoul, Republic of Korea; 4 Department of Electrical Engineering at Korea University, Seoul, Republic of Korea

**Keywords:** Electrical and electronic engineering, Mechanical engineering

## Abstract

The desire to directly touch and experience virtual objects led to the development of a tactile feedback device. In this paper, a novel soft pneumatic actuator for providing tactile feedback is proposed and demonstrated. The suggested pneumatic actuator does not use an external air compressor but it is operated by internal air pressure generated by an electrostatic force. By using the actuator, we designed a glove to interact with virtual reality. The finger motions are detected by attached flexible piezoelectric sensors and transmitted to a virtual space through Bluetooth for interconnecting with a virtual hand. When the virtual finger touches the virtual object, the actuators are activated and give the tactile feedback to the real fingertip. The glove is made of silicone rubber material and integrated with the sensors and actuators such that users can wear them conveniently with light weight. This device was tested in a virtual chess board program, wherein the user picked up virtual chess pieces successfully.

## Introduction

**Figure 1:**
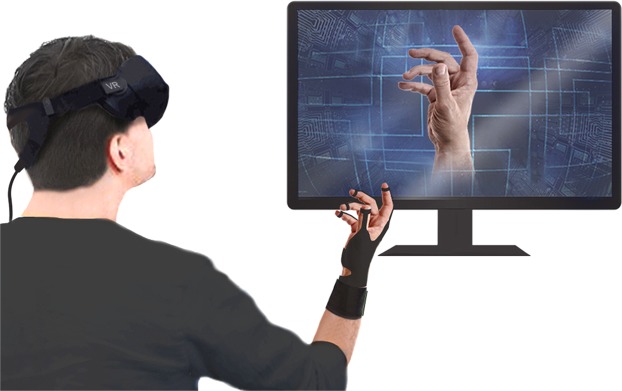
A glove for user interaction with VR. The glove transmits the hand motion of the user to the VR and transmits the stimulus to the user. The figures were created by the authors.

In order to directly experience and feel the virtual reality (VR), various technologies connecting VR and the real world have been developed^1–3^. Head-mounted displays for the surrounding view and gloves for hand motion recognition and tactile feedback are typical examples^4–9^. In particular, the human-computer interface gloves are essential devices for the users to experience the VR by conveying the user’s movements to the VR and transmitting the tactile feedback to the user^10^ (Fig. 1). Using this glove, the user can grab or place objects in VR and can feel the textures of virtual objects^11–13^. In addition, the gloves can be used as text input device^14^. For performance improvement, the interface gloves have been reported to be using various methods and materials including inertial measurement sensors, potentiometer-based sensing technique, or piezoresistive sensor^15–19^. Furthermore, for more practical and comfortable usage, delicate interactive gloves based on novel materials and structures are being developed.

The core elements of the gloves are largely divided into sensors and actuators. The sensors detect the movements of the users and send the motion information to the VR^20^. Among the various sensing materials, piezoelectric materials can be a good candidate for human-computer interaction^21–24^. The piezoelectric materials are either embedded in the gloves as a sensor for hand motion recognition or in an energy harvester using the motion^25–27^. Flexible piezoelectric sensors have a few tens of micro-scale thickness, making them easy to mount on wearable devices^28–30^. In this study, we select polyvinylidene fluoride (PVDF) as a piezoelectric material for the sensor. PVDF materials have also found the applications in actuators, and energy harvesters^31–34^. When the PVDF sensor is bent, we can measure voltage output from the sensor, analyze the value, and estimate the bending shape^35,36^. In our previous study, we tested and validated the PVDF sensing ability to detect the changes in the finger joints: the sensor outputs were compared with the real angles obtained from the camera recording images, and they matched well^36^.

Various actuators have been developed and installed for tactile feedback^37–39^. Actuators to provide mechanical stimuli are more commonly used because they can accurately reproduce the actual texture^40^. They are found in cell phones and pagers and can provide information about the contact force, texture, and roughness of an object. However, the main limitation of vibration tactile stimulation and lateral strain stimulation is that the actuator cannot provide information about the actual surface shape of the object^41^. In addition, mechanical actuators that require large systems are problematic in terms of weight and portability^42^. Soft actuators that provide smooth and flexible tactile feedback can be an alternative to address those problems^43–50^. The soft actuators have various functional advantages, including their light weight and flexibility^48,51^. Because the soft actuators are usually made of flexible materials such as polymers, they have a high strain density and are easy to fabricate as per the desired shape^52,53^. In addition, flexible actuators with relatively simple mechanisms perform multiple degrees-of-freedom motions that can be handled by complicated control systems and large-scale components of hard machines^54–57^. Owing to its advantages, soft actuators have already been utilized in various fields, including medical and wearable applications^44,53^. Thus, herein, we developed and utilized a soft pneumatic actuator (SPA) for tactile feedback. Pneumatic actuators have advantage of light weight, simple system, high speed, and miniaturization^58–62^. However, they need air pressure provided by an external compressor. Because of the existence of the compressor, the entire system using the pneumatic actuators can be bulky. Notably, our actuator uses the internal air pressure generated by an electrostatic force, without an external air compressor. To obtain the internal air pressure, the flexibility of the actuator is very important.

For flexibility, we fabricated the actuator with silicone rubbers. Silicone rubber has an average modulus of elasticity of several hundred kPa, a Poisson’s ratio close to 0.50, and a shear modulus of several tens kPa^63,64^. In case of Ecoflex, a commercial silicone rubber, its elastic modulus is 125 kPa^63^. Additionally, because silicone is harmless to the human body, soft or porous silicone is used for rehabilitation, wearable application, as a surgical material, and in daily life^65–67^. Therefore, Ecoflex can be used as an actuator that touches the human body directly.

To sum up, in this paper, human-computer interface glove system with sensors and actuators is fabricated as one-body. Without additional equipment, this glove senses and transmits hand movements and provides haptic feedback. The mounted actuator is flexible and provides very fast reaction rates. Also, we show the performance test of the glove used in VR.

## Pneumatic Soft Actuator

We developed a new type of pneumatic soft actuator activated by an electrostatic force. The actuator has a small size to give a fingertip tactile feedback. The size and weight are as follows: diameter: 15 mm, height: 5 mm, weight: 0.57 g.

### Operation mechanism

**Figure 2:**
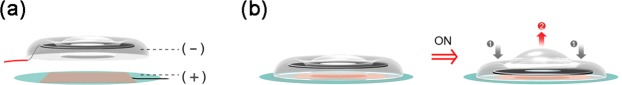
Actuator’s operating mechanism. The figures were created by the authors. (**a**) Different electrodes are connected to the inner electrode of the actuator and the bottom electrode of the copper tape film to induce an electrostatic attractive force. (**b**) The on-off state of the actuator and its operation mechanism.

The actuator can be divided into a ring part, where the electrostatic attractive force works, and the center part, which is the contact part. The silicone thickness of the ring part more than that of the center part (ring: 500 μm, center: 200 μm). When different polarity voltages are applied to the ring part and bottom electrode (Fig. 2a), the ring part moves downward by electrostatic attraction (Fig. 2b-1). As the actuator is sealed, the air in the ring part moves to the center, and the central silicone expands and rises upward (Fig. 2b-2). The fingertip of a user can sense this swollen silicone, that is, the tactile feedback.

### Actuator motion tracing

The motion of the actuator was detected in real time with a high-speed 2D laser scanner (LJ-V7001, Keyence, Japan). Data were collected through 1000 line scans per second.

In order to give a variety of tactile feedback while holding the virtual objects, a wide range of movements should be possible. The on/off switching of the actuator at the moment of catching or releasing a virtual object must be fast and accurate. Moreover, it should remain “on” for holding the virtual object. In the same context, we changed the on/off frequency of the actuator from 0.2 Hz to 1 Hz. The input waveform is a square wave, and the peak-to-peak value of the input voltage is 6 kV.

**Figure 3:**
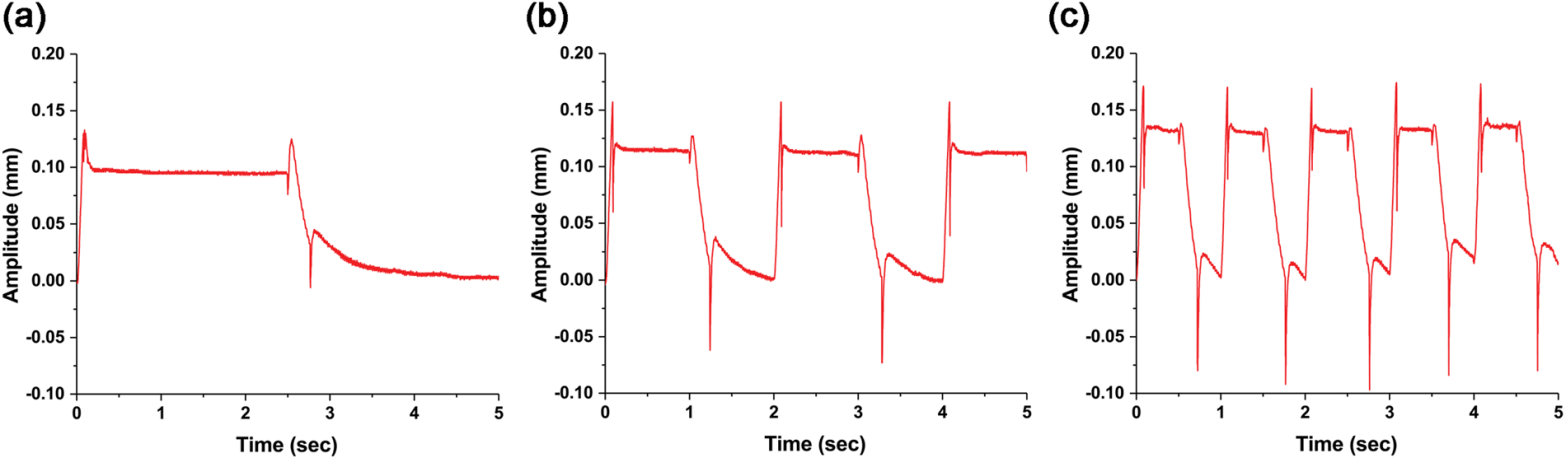
Change in the actuator center displacement according to the input frequency. (**a**) 0.2 Hz. (**b**) 0.5 Hz. (c) 1 Hz.

Figure 3 displays the displacement at the center of the actuator along with the frequency. The displacements are well maintained during the each on/off cycle, although it is observed damping phenomenon by elasticity during the switching time. The peak-to-peak displacements at 0.2 Hz (Fig. 3a), 0.5 Hz (Fig. 3b), and 1 Hz (Fig. 3c) are about 0.10 mm, 0.12 mm, and 0.13 mm, respectively, that is, the displacement increases as the frequency increases. We note that the actuator has the ability to provide enough tactile feedback during the time of holding a virtual object with the reaction speed under a few hundreds of milliseconds.

**Figure 4:**
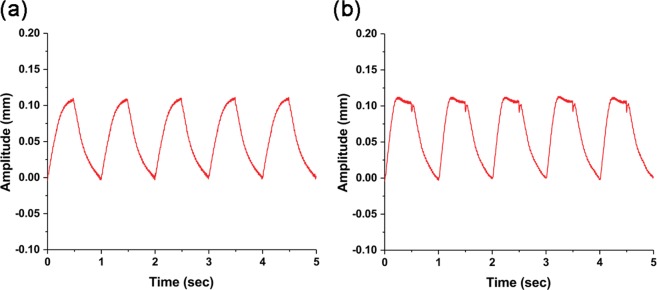
Displacement change of the actuator center according to the input voltage change. (**a**) 0–2.4 kV. (**b**) 0–3 kV

Moreover, the displacement can be varied by the input voltage amplitude (see Fig. 4). The square waves with different voltage levels at 1 Hz were applied. When the input voltage is 2.4 kV, the displacement is about 0.11 mm (Fig. 4a). However, even though the input power is a square wave, the amplitude change seems like a triangle wave. This may be because the input voltage is insufficient to follow the square wave input. When the power source was 3 kV, it presented a square wave such as the input voltage (Fig. 4b). The amplitude is about 0.11 mm. Finally, when the square input is 6 kV, the amplitudes increase to about 0.13 mm and the on state is well maintained (Fig. 3c). We conclude that the actuator is clearly controlled and the amplitude increases as the voltage difference of the input source increase.

## VR Glove With Pneumatic Actuator and Sensor

**Figure 5:**
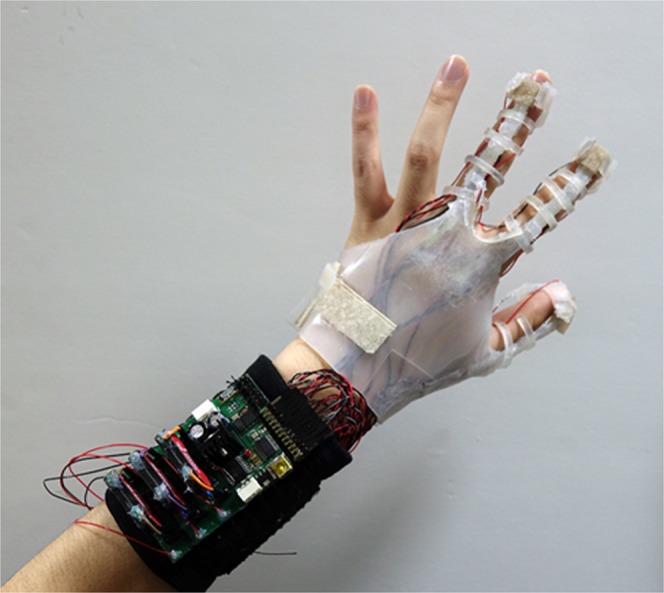
The actual appearance of the integrated glove.

We designed an integrated glove system to work in VR, including the proposed pneumatic actuator (Fig. 5). Specifically, this glove system is divided into two parts, i) hardware part with a flexible glove including piezoelectric sensors, actuators, and interface board, and ii) software part with an interaction system between the real world and VR. In the hardware part, the sensors in the flexible glove collect the joint data, and the interface board transfers the data to the computer system. When the virtual hands touch a virtual object in the interaction system, the “on” signal is sent to the interface board. In the board, the high voltage converter is turned on, and the driving voltage is sent to the actuator. The program in the computer system converts the raw sensor data into finger joint angles for generating hand motions in the VR environment. The total weight of VR gloves including the actuator, sensor, board, and battery, is about 156.2 g.

### Total system with VR

**Figure 6:**
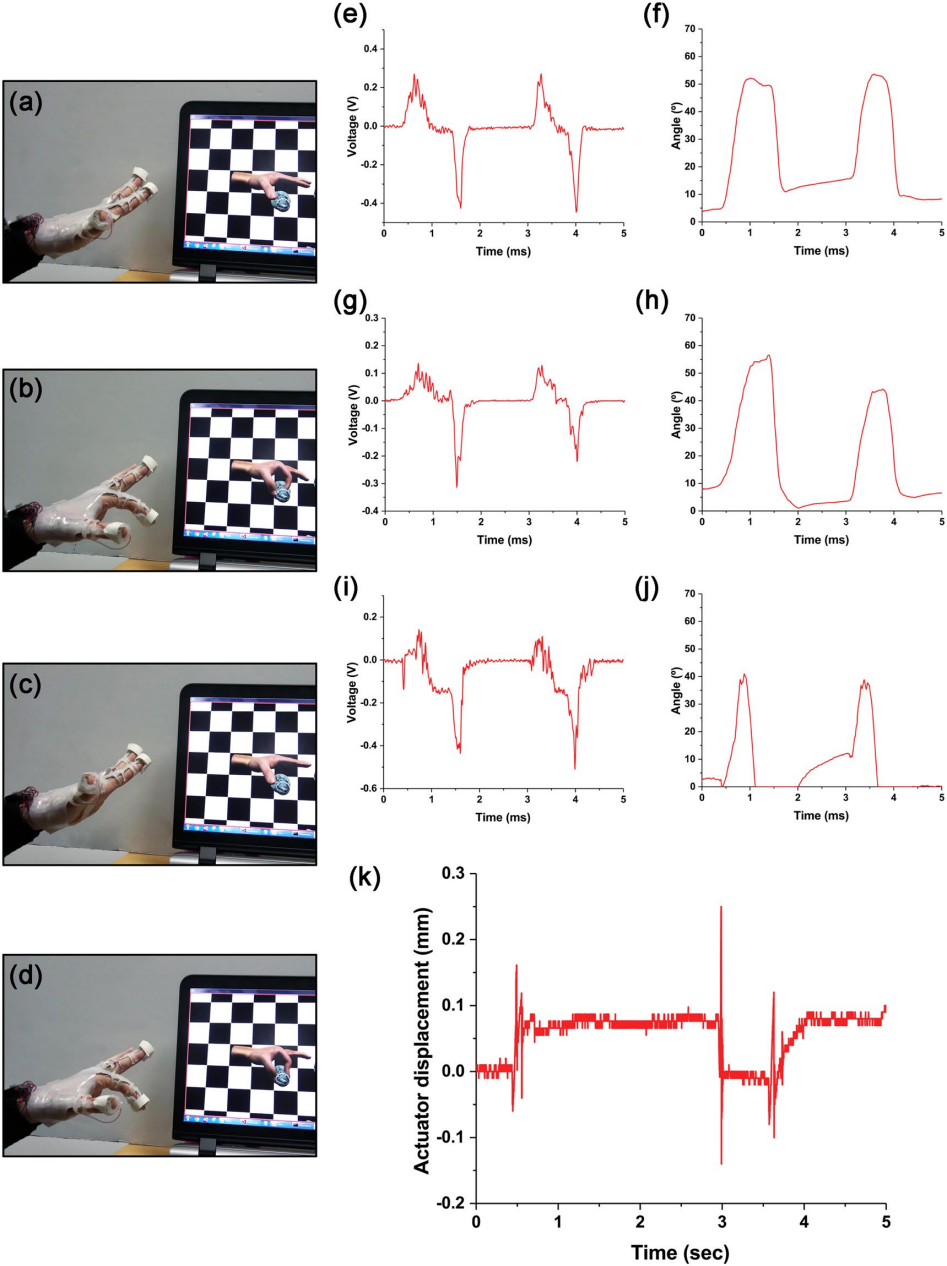
Actual operation of integrated glove for sensor and actuator. (**a**–**d**) Detects the movement of the finger and moves the same in the VR and grabs a virtual object. (**e**) The voltage change and (f) the processed angle by sensing from S1. (**g**) The voltage change and (**h**) the processed angle by sensing from S2. (**i**) The voltage change and (**j**) the processed angle by sensing from S3. (**k**) The amplitude variation of the actuator. The actuator was turned on at the moment of holding the object, and turned off when the object is released.

In this experiment, we used a virtual hand in a VR chessboard to capture a chess horse (Fig. 6a–d)^68^. The finger movements were detected by the sensors and the data were transferred to the program, and the virtual hand of the screen moved based on the data (Fig. 6e–j). Specifically, when the index finger was bent, the sensors gave the changed voltage signals as the output (Figs 6e (S1), 6g (S2) and 6i (S3)). The sensor signals were sent from the glove to the computer, and then processed through time integration and gain correction to obtain the angle sensed from the hand movements^36^.

In particular, the detailed calculation processes for the angle acquirement were as follows.

1 $$\theta ({t}_{n})=A\cdot \sum _{m=1}^{n}(V({t}_{m})-O({t}_{m}))\times {\rm{\Delta }}{t}_{m},n=1,2,\cdots )$$

where $\theta ({t}_{n})$ is a processed angle in *t*_*n*_, ${t}_{n}={\sum }_{m=1}^{n}{\rm{\Delta }}{t}_{m}$ is the system time, *V*(*t*_*n*_) is the voltage value of sensor in *t*_*n*_, and *A* is the gain. Δ*t*_*m*_ is the time interval of the m-th step, and *O*(*t*_*n*_) is the offset voltage of the system in *t*_*n*_. We integrated the value of the difference between the sensor output voltage and offset voltage by the time to obtain the real angles from the sensor outputs. Herein, the integral is to the numerical sum of a rectangular area with the difference value and time interval. Using it, we obtained the offset voltage of the raw sensor output.

2 $$O({t}_{n})=\{\begin{array}{c}\frac{{\sum }_{m=n-{s}_{n}-19}^{n-20}V({t}_{m})}{{s}_{n}},\,20 < {s}_{n} < 120\\ \frac{{\sum }_{m=n-119}^{n-20}V({t}_{m})}{100},\,{s}_{n}\ge 120\\ O({t}_{s}),\,{s}_{n}\le 20\end{array}$$

where *s*_*n*_ is the count value for the non-moving state and O(*t*_*s*_) is the last offset value at the non-moving state. The offset of the sensor was determined with an average of most recent 100 non-moving data. Each 20 data from the start time and end time of non-moving state were excluded for improving the accuracy of the offset value and rapid reaction. To discriminate the moving state, we calculated the non-moving count value and used it as the criterion of the offset calculation, that is, *s*_*n*_. The decision of the moving state was based on the quantity of voltage change. If the voltage change was over a threshold value, *V*_*th*_, we considered the sensor as moving. When the count value was under 20, it was considered as a gap between the moving states, and we used the offset at the last non-moving state. The count value for the non-moving state in *t*_*n*_ is stated as follows,

3 $${s}_{n}=\{\begin{array}{c}0,|V({t}_{n})-V({t}_{n-1})|\ge {V}_{th}\\ \min \,(120,({s}_{n-1}+1)),|V({t}_{n})-V({t}_{n-1})|\le {V}_{th}\end{array}$$

Finally, the gain for estimating the real angle value was multiplied in the calculated value. The gain is obtained by the preliminary calibration operation, and all sensors have different gains^36^.

In this experiment, as shown in Fig. 6, the gain values of S1, S2, and S3 are 657.4 deg/V·s, 1151.0 deg/V·s, and 1843.2 deg/V·s, respectively, and the threshold voltage is 0.05 V. To implement the tactile feedback in the interaction system between the VR and real hand, we transferred the active signal to the control voltage of AGH-60P in the interface board when the virtual hand touches the chess horse.

To show whether the actuator was working at the time of holding the virtual object, an additional actuator that receives the same control signal as that of the actuator of the index finger was setup, and the movement was measured with a line laser sensor. As a result, the virtual hand followed well according to the movement of the hand, and the actuators also operated normally when the hand reached the virtual object (Fig. 6k). Depending on the contact between the hand and the object, the actuator maintained the on/off state and gave the tactile feedback to the user. Supplementary Video 1 shows this experiment.

## Conclusion

In this study, we developed a new SPA and applied it as a glove system that interacts with the VR. The pneumatic actuator has an advantage that it can be operated without an external air compressor. We performed a series of tests using the actuator showing that it can be adjusted periodically. Also it can be attached to the gloves to generate effective tactile feedback. In particular, when the user holds a virtual object, the actuator is well maintained in the on state, and when the virtual object is released, the actuator is switched to the off state. The actuator is actuated by electrostatic attraction. When the air space is reduced by the electrostatic attraction, the central part expands and is designed to give haptic feedback. Especially, the actuator showed a larger movement as the period became faster and the applied voltage became larger. In addition, the designed silicone monolithic glove was able to detect movement of fingers with the PVDF sensors and transmit data via Bluetooth. A voltage output by a piezoelectric sensor deformation provides finger motion information. In order to distinguish the moving state from the received information, the threshold value was specified, and the gain value was obtained through the initial calibration. We expect that our developed glove will be used in several ways by linking with various VR software.

## Methods

### Actuator fabrication method

Fabrication molds for the SPA were designed using Solidworks software (Dassault Systems Solidworks Corp., USA) (Fig. S1a). Then, the design was realized by the main part (VisiJet M3 Crystal, 3D Systems Inc., USA) and supporter (VisiJet S300, 3D Systems Inc., USA) materials in a 3D printer (ProJet HD3500, 3D systems Inc., USA) (Fig. S1b). After printing, the mold was heated in a convection oven (DCF-31-N, Dae Heung Science, Korea) for melting the supporter material. Finally, the melted supporter was completely removed from the mold in an oil bath in an ultrasonic cleaner (Sae Han Ultrasonic Co., Korea). After washing and drying, a release agent (Ease release 200, Smooth-On, Inc., USA) was sprayed on the mold surface to prevent the silicone from sticking to the mold.

After manufacturing the mold manufacture, the silicone was fabricated as the exterior of the actuator (Fig. S1c). First, Ecoflex 0030 part A (Smooth-On, Inc., USA), Ecoflex 0030 part B (Smooth-On, Inc., USA), and platinum silicone cure accelerator (Plat-cat, Smooth-On, Inc., USA) were mixed in a ratio of 1:1:0.04 (Fig. S1d). The well-mixed mixture was poured into the mold and cured at room temperature for 2 h. The specimen with a ring shape was carefully separated from the mold using tweezers after fully curing. Then, we inserted a hemispherical mold into the center of the silicone ring and poured the silicone mixture once more (Fig. S1e). It created a different thickness of the ring and center of the actuator. The hardened silicone was removed from the mold (Fig. S1f). The coiled wire and a carbon conductive adhesive tape (Nisshin EM Co., Ltd., Japan) with a hole in the center were attached to the side of the ring silicone body of the actuator (Fig. S2a). Then the polyethylene terephthalate (PET) film (Saehan, Korea) was attached to the bottom of the silicone body and sealed well. Finally, we attached a carbon conductive tape to the bottom of the PET film as an electrode. A photograph of the completed actuator is shown in Fig. S2b.

A high-voltage converter (AGH 60P-5, XP Power, Singapore) providing an output of 6 kV was utilized for operating the actuator. The high-voltage converter was connected to a power supply (MK3003P, MK power, Korea), and its control pin was connected to a waveform generator (33500Bseries, Keysight technologies, USA), which could output square waves. A thick-film resistor (50 MΩ, Ohmite, USA) was connected between the output pins of this converter for discharging. The (+) and (−) ports of the high-voltage converter were connected to the copper tape at the bottom and the wire in the actuator (Fig. 2a).

### Fabrication method for soft virtual reality glove system

A piezoelectric film (28 µm PVDF Silver Ink, Measurement Specialties, Inc., USA) was cut to sizes of (length) 20 mm × (width) 5 mm for S1–S6/S9 - S11, (length) 30 mm × (width) 5 mm for S8, and (length) 40 mm × (width) 5 mm for S7. The capacitances of the 20 mm, 30 mm, and 40 mm length sizes of the piezoelectric sensors were measured using a graphical sampling multimeter (DMM7510, Keithley Instruments Ltd., USA), and the values were 0.36 nF, 0.54 nF, and 0.70 nF, respectively. Two 2 mm × 5 mm copper tapes (1181, 3 M, USA) were attached to the top and bottom of the sensor and soldered to 0.7 mm diameter electric wires.

A total of 11 sensors were attached to the glove, to detect the movements of the thumb, index finger, and middle finger, with a silicone adhesive (Sil-Poxy, Smooth-On, Inc., USA) (Fig. S3a): sensors attached for collecting distal interphalangeal joint angle, proximal interphalangeal joint angle, metacarpophalangeal joint angle of the thumb, index finger, and middle finger, and abduction/adduction angle between the fingers.

We also added three pneumatic actuators (A1–A3) to provide tactile feedback on the tips of the thumb, index finger, and middle finger (Fig. S3).

In particular, the silicone-based glove was fabricated by using 3D-printed molds (Figs S4a and S4b). The silicone was poured into the printed molds (Fig. S4c). After curing, the glove contour fabrication was completed (Fig. S4d). Moreover, the holders for the actuator attachment to the glove were also made using silicone (Fig. S4e). The rings were 3D-printed for the glove to wear on the fingers (Fig. S4f). The holders and rings were attached to the glove using the silicone adhesive.

To measure the sensor output, control the actuators, and communicate with the computer, we utilized the interface board as shown in Fig. S5a. The board size was 80 mm × 55 mm, including ATMEGA328P-AU as the main microcontroller and F1E22 as a Bluetooth module. For the actuator control, three output nodes transmitted on/off values through the signal isolator into the control voltage pin of the high-voltage amplifier EMCO AGH-60P (Fig. S5b). For the sensor measurement, the microcontroller collected the voltages from the 11 sensors through the internal 10-bit analog–digital converter. We utilized an analog multiplexer because of the limitation of the analog pins. In particular, three sensors were directly connected to the analog pins in the microcontroller, and other sensor outputs were measured through the multiplexer (Fig. S5c). Each sensor was connected with a 10 MΩ load resistor, and one electrode was connected with +2.5 V, produced from the voltage regulator SPX1587AU-2.5. During data processing of the sensor output, a 30-Hz low-pass filter was used to attenuate the 60 Hz noise from the power sources.

Soft virtual reality glove system was worn by one of the author and we agreed to publish the identification information, images and videos included in this study.

## Supplementary information

Figures S1,S2,S3,S4, and S5

VR glove test movie
