# Supplementary material for: Pneumatic actuator and flexible piezoelectric sensor for soft virtual reality glove system
Source: Sci Rep. 2019 Jul 18;9:8988. doi: 10.1038/s41598-019-45422-6 (PMC6639318; doi:10.1038/s41598-019-45422-6)
Supplement: Supplementary file 1 — Figures S1,S2,S3,S4, and S5 [file 41598_2019_45422_MOESM1_ESM.docx]

**Pneumatic actuator and flexible piezoelectric sensor for soft virtual reality glove system**

**Kahye Song^1^, Sung Hee Kim^1^, Sungho Jin^2^, Sohyun Kim^3^, Sunho Lee^4^, Jun-Sik Kim^1^, Jung-Min Park^1^ and Youngsu Cha^1^***

^1^ Center for Intelligent & Interactive Robotics, Korea Institute of Science and Technology, Seoul, Republic of Korea

^2^ Department of Biomedical Engineering at Korea University, Seoul, Republic of Korea.

^3^ Department of Mechanical System and Design Engineering at Seoul National University of Science and Technology, Seoul, Republic of Korea.

^4^ Department of Electrical Engineering at Korea University, Seoul, Republic of Korea.

**Corresponding author*: Y. Cha**

Phone: +82-2-958-6949; e-mail: givemong@kist.re.kr.


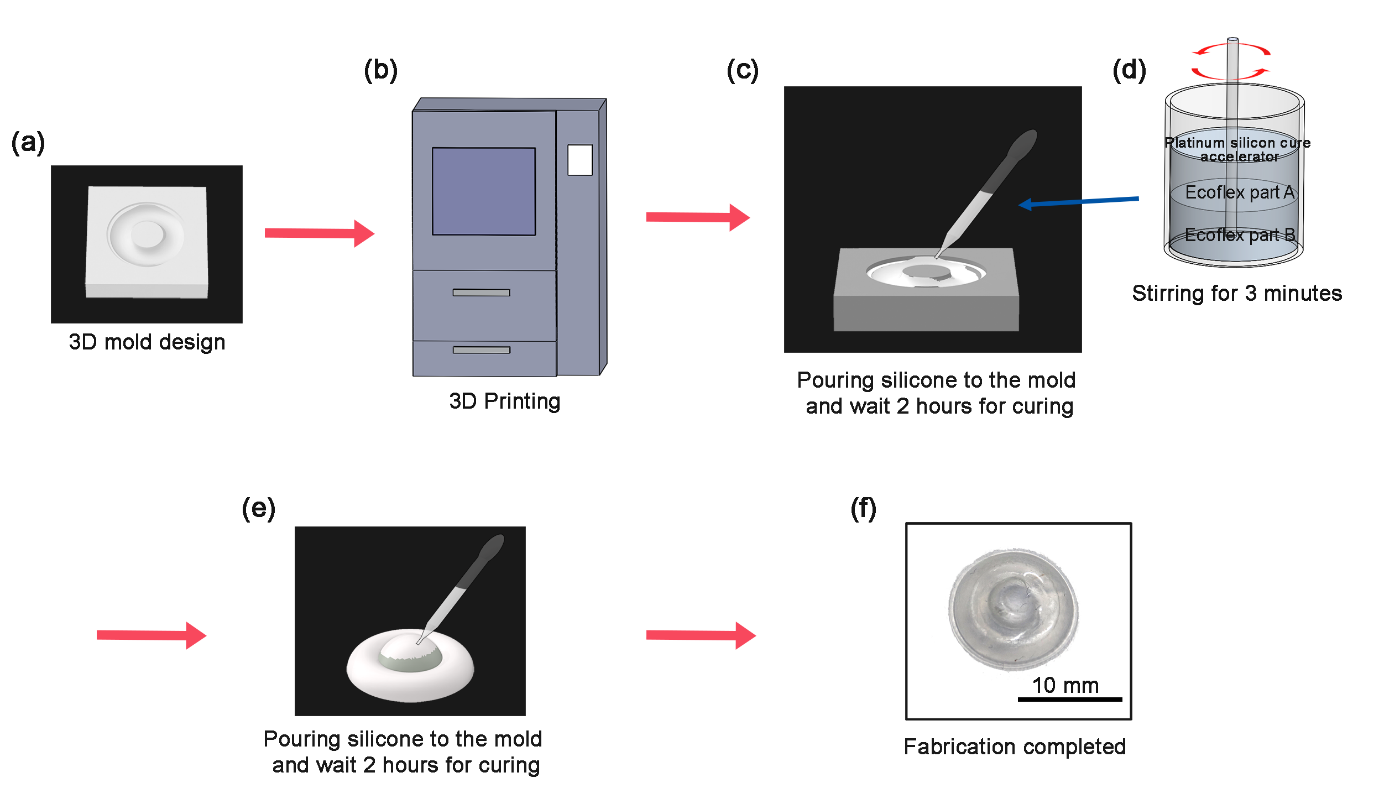


**Supplementary Figure S1.** Actuator fabrication method. The figures were created by the authors. (a) Mold designed in 3D. (b) 3D printing. (c) A state in which silicone is poured into the completed mold. (d) Blending ratio for silicone fabrication (e) Silicone treatment to create a central shape. (f) Completed actuator geometry.


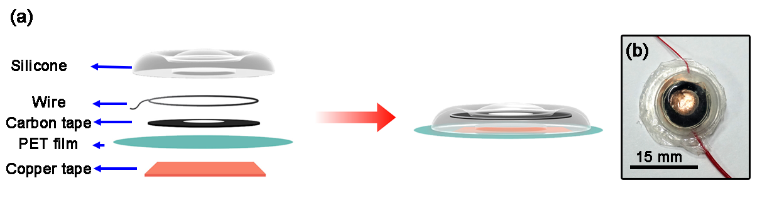


**Supplementary Figure S2. (**a) Internal structure of the actuator. The carbon film and wire for electrodes are built in. A PET film to prevent electrical shorts seals the inside air with silicone. The figures were created by the authors. (b) Photo of the fabricated actuator.


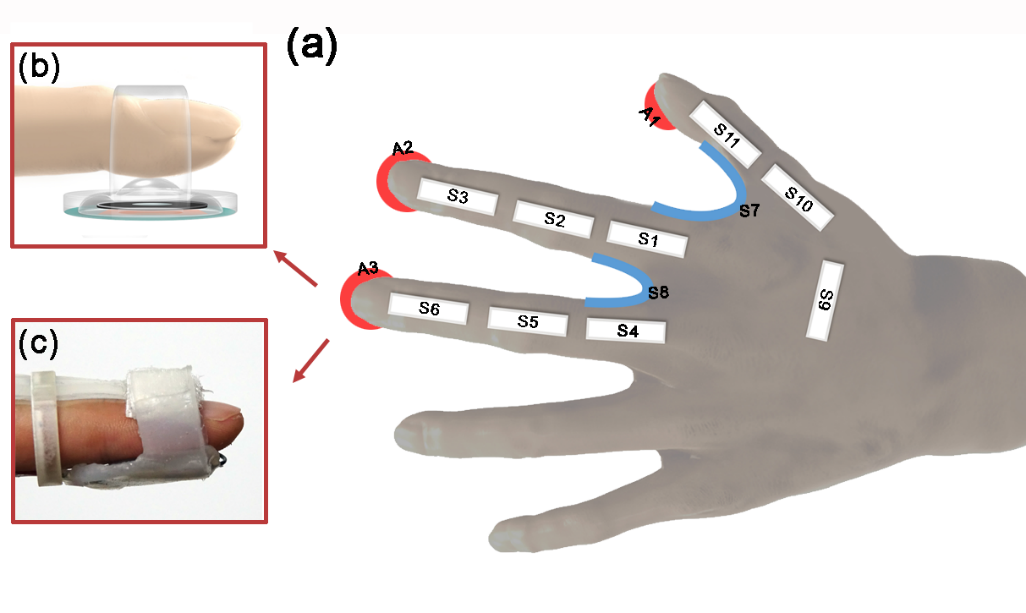


**Supplementary Figure S3.** (a) Schematic for positioning the actuators and sensors inside the integrated glove. The figures were created by the authors. (b) Schematic for the mounted actuator on the fingertip. (c) Photo of an actuator on a real fingertip.


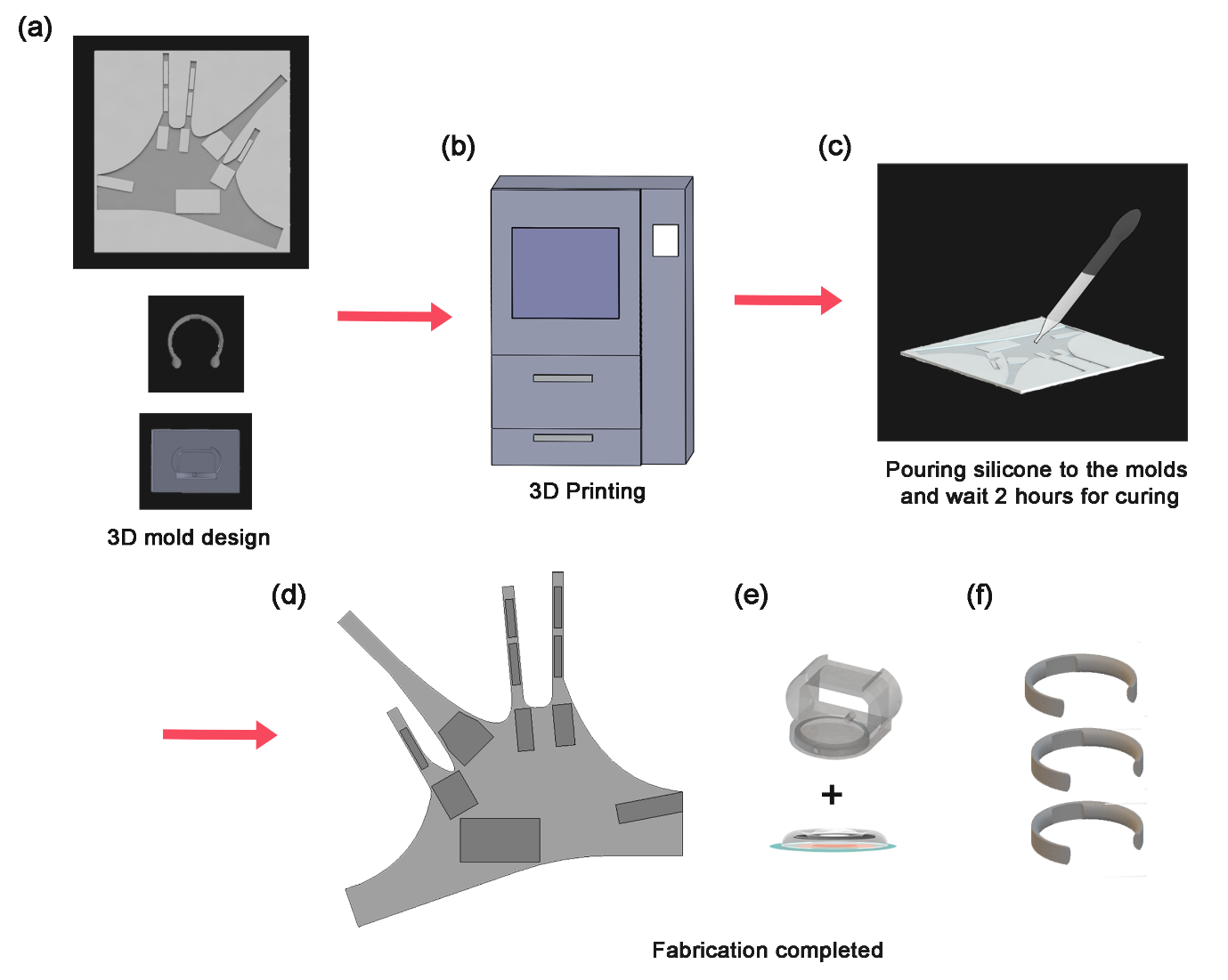


**Supplementary Figure S4.** Integrated gloves and accessories fabrication method. The figures were created by the authors. (a) Mold designed in 3D. (b) 3D Printing. (c) A state in which silicone is poured into the completed mold. (d) Silicone glove contour. (e) Holder for the actuator attachment. (f) Finger rings.


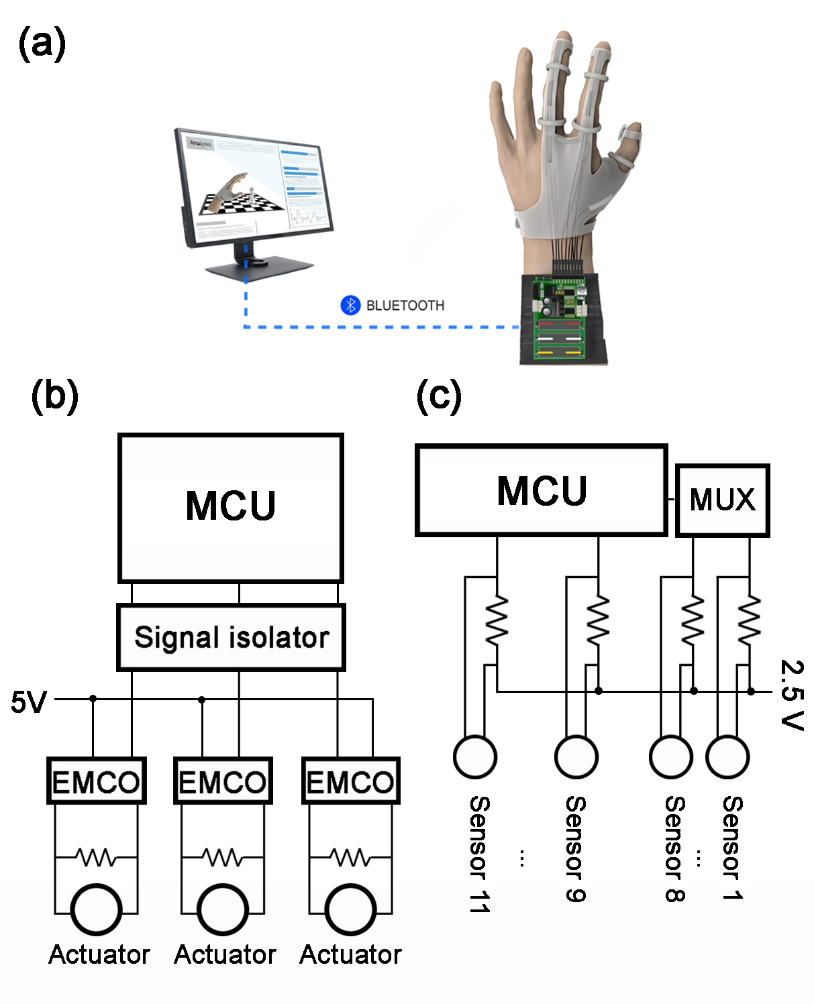


**Supplementary Figure S5.** (a) A schematic of the VR to communicate with the integrated glove for the sensors and actuators. The figures were created by the authors. (b) Circuit diagram for the actuator operation. (c) Circuit diagram for the sensor measurement.
